# Accommodating cognitive screening: An implementation study protocol for occupational therapists supporting clients with vision and/or hearing impairments

**DOI:** 10.1002/alz.086242

**Published:** 2025-01-09

**Authors:** Shirley Dumassais, Walter Wittich

**Affiliations:** ^1^ Université de Montréal, Montreal, QC Canada; ^2^ École d'optométrie, Université de Montréal, Montréal, QC Canada

## Abstract

**Background:**

Occupational therapists (OTs) play a crucial role in rehabilitation, aiming to improve the lives of individuals with sensory impairment and an increased risk of cognitive decline. Despite being responsible for cognitive screenings on occasion, they have expressed limited confidence in performing these with their clients with visual and/or hearing impairments (VI/HI). To improve this situation, OTs should gain expertise in collaborative sensory rehabilitation. The primary aim of this multiphasic implementation study is to advance rehabilitation, particularly in occupational therapy, by enhancing the proficiency, knowledge, and skills of practicing clinicians when administering cognitive tests to individuals with sensory impairment(s).

**Methods:**

This study employs a comprehensive five‐phase methodology, starting with (1) a scoping review to identify existing practices in the research context. Subsequently, (2) a clinical practice survey explores current approaches within the field of occupational therapy. (3) Interviews and focus groups with OTs provide further qualitative insights. Then, (4) educational training resources are developed, and (5) their efficacy is assessed in the final phase, ensuring their quality, application, and perpetuation.

**Results:**

Presently, the first two phases of this project have been completed and the third phase is underway. In the scoping review, the identified common strategies s in the cognitive evaluation of older adults with dual sensory impairment (DSI) include expert team involvement, using visual‐free cognitive tests, modifying scoring procedures, and implementing communication and environmental strategies. In the survey, OTs indicated customizing accommodations for individuals with VI and/or HI. Strategies included encouraging the use of visual aids and adjusting lighting for VI, promoting hearing aids and controlling noise for HI, and employing diverse approaches for DSI, such as subjective screening and recommending assistive technology.

**Conclusions:**

Building on evidence‐based research as well as current practices, the collaboration between specialists and clinicians will bridge the knowledge‐to‐practice gap in rehabilitation through accessible education. Empowering OTs with such knowledge in their practice has the potential to improve the process of cognitive screenings as well as healthcare outcomes.

